# Effects of high-frequency transcranial magnetic stimulation on functional performance in individuals with incomplete spinal cord injury: study protocol for a randomized controlled trial

**DOI:** 10.1186/s13063-017-2280-1

**Published:** 2017-11-06

**Authors:** Amanda Vitória Lacerda de Araújo, Valéria Ribeiro Nogueira Barbosa, Gilma Serra Galdino, Felipe Fregni, Thais Massetti, Sara Lynn Fontes, Danilo de Oliveira Silva, Talita Dias da Silva, Carlos Bandeira de Mello Monteiro, James Tonks, Fernando Henrique Magalhães

**Affiliations:** 1 School of Arts, Sciences and Humanities, EACH – USP, University of São Paulo, São Paulo, Brazil; 2 Department of Physiotherapy – UEPB, Paraíba State University, Campina Grande, Brazil; 3 Harvard Center for Noninvasive Brain Stimulation, Harvard Medical School, Boston, MA USA; 4 Faculty of Medicine, University of British Columbia, Vancouver, BC Canada; 5 Physical Therapy Program – UNESP, State University of São Paulo, São Paulo, Brazil; 6 University of Exeter Medical School, Exeter, UK; 7 University of Lincoln, Lincoln, UK; 8 Haven Clinical Psychology Practice, Cornwall, UK; 9 Biomedical Engineering Laboratory, Department of Telecommunication and Control, Avenida Professor Luciano Gualberto, Travessa 3, n. 158. Cidade Universitária, São Paulo, SP 05508-010 Brazil

**Keywords:** Incomplete spinal cord injury, Plasticity, Motor rehabilitation, Non-invasive brain stimulation, Repetitive transcranial magnetic stimulation

## Abstract

**Background:**

Repetitive transcranial magnetic stimulation (rTMS) has been investigated as a new tool in neurological rehabilitation of individuals with spinal cord injury (SCI). However, due to the inconsistent results regarding the effects of rTMS in people with SCI, a randomized controlled double-blind crossover trial is needed to clarify the clinical utility and to assess the effect size of rTMS intervention in this population. Therefore, this paper describes a study protocol designed to investigate whether the use of rTMS can improve the motor and sensory function, as well as reduce spasticity in patients with incomplete SCI.

**Methods:**

A double-blind randomized sham-controlled crossover trial will be performed by enrolling 20 individuals with incomplete SCI. Patients who are at least six months post incomplete SCI (aged 18–60 years) will be recruited through referral by medical practitioners or therapists. Individuals will be randomly assigned to either group 1 or group 2 in a 1:1 ratio, with ten individuals in each group. The rTMS protocol will include ten sessions of high-frequency rTMS (5 Hz) over the bilateral lower-limb motor area positioned at the vertex (Cz). Clinical evaluations will be performed at baseline and after rTMS active and sham.

**Discussion:**

rTMS has produced positive results in treating individuals with physical impairments; thus, it might be promising in the SCI population. The results of this study may provide new insights to motor rehabilitation thereby contributing towards the better usage of rTMS in the SCI population.

**Trial registration:**

ClinicalTrials.gov, NCT02899637. Registered on 25 August 2016.

**Electronic supplementary material:**

The online version of this article (doi:10.1186/s13063-017-2280-1) contains supplementary material, which is available to authorized users.

## Background

Spinal cord injury (SCI) affects about 2.5 million individuals worldwide and often leads to severe disability, due to functional limitations in the sensory and motor systems [1]. After trauma or pathological processes upon the spinal cord, there is usually some preservation of sensation, or motor function at the lowest segment of the spinal cord, a condition known as incomplete SCI (iSCI) [2]. iSCI has been associated with a serious reduction in quality of life and functional independence [3, 4]. Therefore, effective rehabilitation programs are required for patients with iSCI in both acute and chronic care.

Processes of neural regeneration and plasticity can result in significant functional recovery after iSCI [5]. Spontaneous recovery of motor and sensory function in iSCI individuals can be substantial, but highly variable [6]. Repetitive transcranial magnetic stimulation (rTMS) rehabilitation techniques, based on protocols that selectively stimulate specific pathways along the central nervous system (CNS), have been found to be effective in enhancing neurological recovery leading to improved functional abilities [7]. rTMS is a procedure that involves repetitively delivering biphasic magnetic pulses over a specific cortical site [8, 9] to provide stimulation of the corticospinal tract (CST), primary motor cortex (M1), and spinal cord, so as to induce neuronal reorganization, which can be largely involved in the control of voluntary movements [10].

Protocols involving rTMS have been used to induce changes in the excitability of neuronal circuits with positive effects [11–16] at the site of stimulation or transsynaptically at distant sites such as spinal cord circuits [17, 18]. The effect of high-frequency rTMS (i.e. ≥ 5Hz) includes changes in synaptic plasticity resembling long-term potentiation (LTP), as well as shifts in network excitability, activation of feedback loops, and activity-dependent metaplasticity [19, 20]. On this basis, rTMS is emerging as a promising technique in improving neurophysiological outcomes and voluntary motor output in patients with motor disorders [11, 21, 22]. Indeed, studies indicate that motor improvements may be due to modification of corticospinal projections by increasing motor cortical excitability [14–16] and, consequently, promote plasticity associated with functional recovery [22]. Additionally, reduction in spasticity may occur through enhancement of descending corticospinal projections and segmental effects on spinal interneurons that might strengthen inhibitory connections [13, 14, 23].

Many studies have used rTMS as a non-invasive and painless method to induce long-lasting changes in the excitability of cortical and corticospinal pathways after iSCI. In this scenario, rTMS has been found effective in enhancing corticospinal synaptic transmission [12], reducing spasticity [13, 14, 23] and improving sensorimotor function after iSCI [14–16], although we note none of these studies reported associated effect sizes in their analyses. However, other studies report less positive findings, including unchanged sensorimotor function [24], as well as unaltered cortical excitability and level of spasticity [16] in response to rTMS. The lack of consistent results is likely associated with differences in stimulation parameters (e.g. intensity, frequency, number of pulses), number of sessions, relative location of M1, chronicity and levels of injury, and outcome measurements used in previous studies [10].

In considering three particular studies that have examined the effects of rTMS in individuals with iSCI, we note interesting results on sensory and motor performance. Belci et al. [15] delivered double pulses of rTMS over the M1 representation of thenar muscles (360 doublets, separated by 100 ms). They used a low frequency (with 10 s between each doublet and stimulus intensity corresponding to 90% of resting motor threshold [RMT]) for five days in a group of four individuals with iSCI. Results indicated that active rTMS improved somatosensory and motor function at three weeks following stimulation, as assessed by the American Spinal Injury Association Impairment Scale (ASIA) nine-hole peg test and electrical perceptual threshold measurements. Additionally, Benito et al. [14] applied 15 sessions of sham or rTMS over the leg representation of M1 (20 trains of 40 pulses at 20 Hz with an intensity of 90% of RMT) in 17 individuals with iSCI. This study found significant improvements in motor function for at least two weeks, as assessed by the ASIA scale for lower limbs and gait function. Contrary to the findings noted above, ASIA scores were unaltered in Kuppuswamy et al. [24]. The protocol had five days of active or sham rTMS (900 pulses at 5 Hz in 2 s trains separated by 80-s intervals, at 80% of active motor threshold) applied to M1 representations of either the hand or forearm muscles in 15 individuals with iSCI. It is worth noting that the studies cited above have reported sham placebo-controlled trials which were single-blinded [15, 24], non-randomized [15], and either all [15, 24], or at least part of the individuals crossed over between groups during the study [14].

Therefore, while there is good evidence for its use in other diseases, the literature regarding the efficacy of rTMS in individuals with iSCI is inconsistent, as the few studies that have addressed this issue yielded contrasting results. Due to the quality of the study design as well as to the differences in rTMS parameters used in previous studies, there is no strong evidence of consistent changes in sensory and motor function in individuals with iSCI after rTMS [10]. Thus, there are gaps in the knowledge base and hence the development of a new prospective study, with a fully randomized double-blind placebo-controlled design [25], is necessary to provide evidence of the clinical utility of rTMS in individuals with iSCI. Due to the positive effects of rTMS in inducing long-lasting changes in spinal and supraspinal circuits, we hypothesized that rTMS applied over the lower-limb motor area will: (1) improve motor function; (2) improve sensory function; and (3) reduce spasticity.

Therefore, our aim was to identify a protocol for a double-blind randomized controlled crossover trial that will be performed to evaluate the effects of high-frequency rTMS on the sensorimotor function and spasticity in individuals with iSCI.

## Methods/Design

We registered this trial on ClinicalTrials.gov (NCT02899637). This paper has been reported in accordance with the Standard Protocol Items: Recommendations for Interventional Trials (SPIRIT) [26].

### Study design

**Fig. 1:**
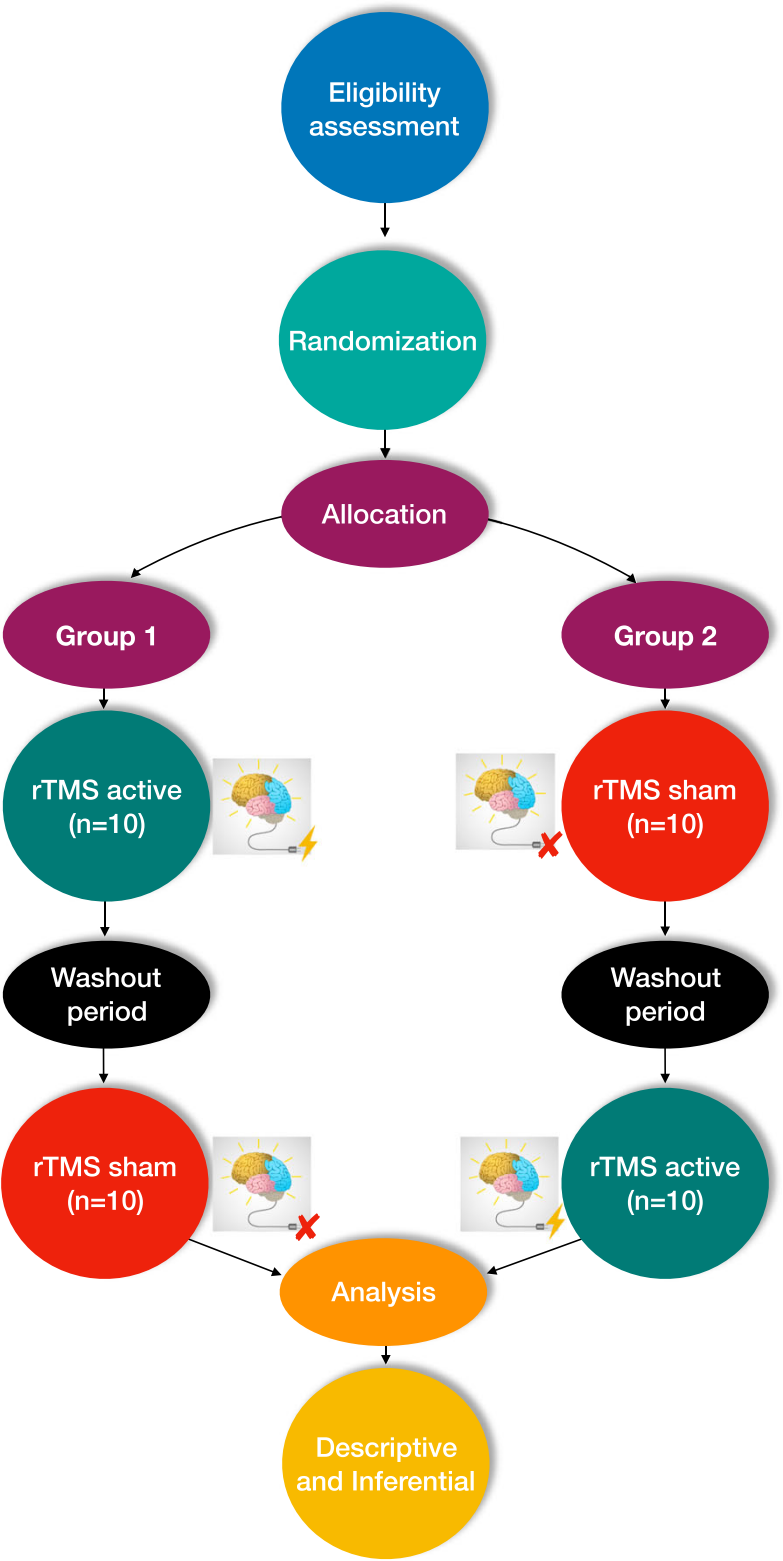
*Flowchart* for the rTMS study protocol. The participants will be selected and the eligibility assessment will be applied. They will then be randomized and allocated to group 1 (starting with rTMS-active) or group 2 (starting with rTMS-sham). Five sessions of rTMS-active and sham will be applied to participants in each group over one week, with a washout period (two weeks). Assessments will be before and after each intervention period (i.e. sham and active) using the motor and sensory scales, spasticity scale, and surface electromyography

**Fig. 2:**
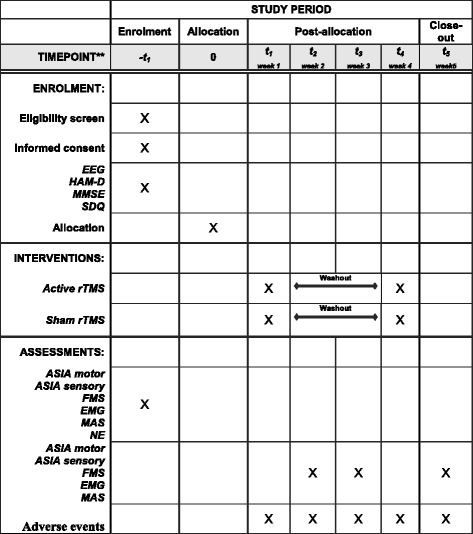
SPIRIT figure. Description of the rTMS study protocol

A double-blinded randomized controlled crossover trial will be conducted and all participants will undertake rTMS-active and sham. Group 1 will start with five sessions of rTMS-active. After two weeks, this group will be reallocated to five sessions with rTMS-sham. In contrast, group 2 will do the opposite protocol (participants will start allocated to five sessions of rTMS-sham, and after a two-week washout period will be reallocated to five sessions of rTMS-active). The two-week washout period has been used in some studies [24, 27] and was shown to be enough to reset the effects of the first five sessions, considering that the motor effects of the rTMS in individuals with iSCI are sustained for 1 h [24] to two weeks [14] and that spasticity effects endure for one week [16, 23]. Figure 1 summarizes the planned experimental design. This research protocol follows the SPIRIT recommendations. For the SPIRIT Checklist see Additional file 1 and for the SPIRIT Figure see Fig. 2. Table 1 provides an overview of the trial characteristics, based on the WHO Trial Registration Data Set.

**Table 1:** Trial characteristics based on WHO Trial Registration Data Set

Data category	Trial information
Primary registry and trial identifying number	ClinicalTrials.gov, ID: NCT02899637
Date of registration in primary registry	25 August 2016 on
Secondary identifying numbers	Ethical Committee of Paraiba State University, under the number CAEE: 18753713.0.0000.5187
Source(s) of monetary or material support	Foundation of Support for Research of São Paulo State - FAPESP #2015/13096-1 and Coordination for Higher Education Staff Development - CAPES.
Primary sponsor	University of Sao Paulo – USP
Secondary sponsor(s)	NA
Contact for public queries	FHM, AVLA
Contact for scientific queries	FHM, AVLA
Public title	Effects of transcranial magnetic stimulation on incomplete spinal cord injury
Scientific title	Effects of high-frequency transcranial magnetic stimulation on functional performance in individuals with incomplete spinal cord injury: study protocol for a randomized controlled trial
Country of recruitment	Brazil
Health condition(s) or problem(s) studied	Spinal cord injury
Interventions	High-frequency repetitive transcranial magnetic stimulation on the lower-limb area of the motor cortex, over one week (five consecutive sessions – once a day)
Key inclusion and exclusion criteria	Inclusion criteria: clinical diagnosis of iSCI with non-progressive etiology; at least six months post iSCI; clinical stability; age range 18–60 years; satisfactory score in Scale of Mini-Mental State Examination (i.e. cut-off points of 13 for illiterates, 18 for low and middle school, and 26 for high school); no pathological alterations on electroencephalography; absence of depression as assessed by the Hamilton Depression Scale and receive sensorimotor conventional physiotherapy. Exclusion criteria: metal prosthesis in some part of the body; cardiac pacemaker; either cognitive impairment, psychotic, or either schizophrenic disorders; neuropsychiatric co-morbidity; drugs that reduce seizure threshold or spasticity
Study type	Interventional allocation: randomized
	Masking: double-blind
	Assignment: crossover
	Primary purpose: treatment
Date of first enrolment	December 2017
Target sample size	20
Recruitment status	Recruiting
Primary outcome(s)	Change in motor scores from baseline to four weeks
Key secondary outcome(s)	Assessment of change in sensory and spasticity scores from baseline to four weeks

*NA* not available

### Participants and sampling

Participants will be recruited through referral by medical practitioners or therapists who work at the Physiotherapy Health Center of Paraíba State University in Brazil in single-stage cluster sampling. Those interested in participating will undergo a detailed screening against the eligibility criteria for enrollment in the study.

### Inclusion criteria

Participants will be included if they have agreed to participate in the study and have signed an informed consent form. They will have a clinical diagnosis of iSCI with non-progressive etiology which is characterized by spinal, vascular, and infectious trauma. Neuroimaging examinations, such as computed tomography and nuclear magnetic resonance imaging, will be used to exclude SCIs due to progressive worsening conditions such as neurodegenerative, tumor, and demyelinating pathologies [28, 29]. The participants should be at least six months post iSCI, clinically stable, aged 18–60 years, have a satisfactory score in Scale of Mini-Mental State Examination (MMSE) (i.e. cut-off points of 13 for illiterates, 18 for low and middle school, and 26 for high school), have no pathological alterations on electroencephalography (EEG), be clear of depression as assessed by the Hamilton Depression Scale (HAM-D), and be currently receiving conventional sensorimotor physiotherapy, to a maximum of three times per week.

### Exclusion criteria

Participants will be excluded if they have a metal prosthesis in some part of the body, use a cardiac pacemaker, have either cognitive impairment, psychotic, or schizophrenic disorders as diagnosed by the rehabilitation team, neuropsychiatric co-morbidity, or use drugs that reduce either seizure threshold or spasticity. Individuals participating in intensive programs of rehabilitation that could bias the results will also be excluded.

EG before the rTMS procedure will identify possible brain bioelectrical abnormalities (such as interictal epileptiform discharge, generalized photosensitivity, burst suppression, hypsarrhythmia) that could be related to the possibility of epileptic discharges [30, 31], thus reducing the risk of seizures during rTMS, since the high-frequency stimulation might lead to cortical hyperactivity. If any of these abnormalities are identified, the individual will be excluded.

### Dropout criteria

Participants will be withdrawn from the study if they are not willing to continue their participation, cannot be present on the day of the experiment or miss a treatment section, and/or change their form of rehabilitation during the study.

### Randomization

Participants will be randomly allocated to either group 1 (rTMS-active) or group 2 (rTMS-sham) with a 1:1 allocation defined by a computer-generated randomization using the R package (R Foundation for Statistical Computing) [32]. Randomization will be under the control of a blinded investigator who will be the only person allowed to manage the electronic security file of the randomization to assign the individuals. The investigator will be blind to the group in which the participant is allocated to.

### Blinding

The participants, researchers, and outcome assessors will remain blind to group allocation during the study. To ensure proper blinding, participants will receive codes and will be concealed from the allocation process by one different researcher. The researchers responsible for applying the intervention and the outcome assessors will not know the study design, allocation, objectives, and expected outcomes.

In addition, for the blinding of the experimenter (responsible for applying the intervention), one assessor (responsible for randomization) will be in charge of giving the active or sham coil to the experimenter. The sham coil has exactly the same shape of the active coil. Further details are presented in the “rTMS-sham” section.

### Allocation concealment

Allocation concealment will successfully be reached since no one involved in this study (i.e. the participants, researchers, and outcome assessors) will be aware of the treatment allocations. Furthermore, investigators will have no control over the order of patients randomized. A blinded investigator will encode the individuals and groups of intervention. To perform the allocation procedure, the encoded groups will be placed inside a closed opaque envelope, which will be labeled with the code for each participant. Envelopes will be opened only during the time of active or sham intervention.

### Intervention

All participants will attend the assigned rTMS intervention as follows: there will be ten sessions over two weeks, in which five sessions will be active and five will be sham, separated by a two-week washout period. The sessions will be administered consecutively and once a day. The researchers will be trained to perform both rTMS-sham and rTMS-active interventions.

#### rTMS-active

Over five consecutive sessions per week (i.e. one session daily), rTMS-active will be performed with a frequency of 5 Hz and 12 pulse trains. The stimulation intensity will be set at 100% of the motor threshold of the area corresponding to the *abductor pollicis brevis*. The stimulation target will be the area associated with the bilateral lower-limb motor area (i.e. vertex, Cz) of the M1.

We chose an intensity of 100%, based upon the limitations reported by Kumru et al. [16], who showed that an intensity of 90% applied during active rTMS was relatively low for leg muscles. In this line of reasoning, Rossini et al. [17] suggests that stronger descending excitatory drive shall be obtained by higher stimulus intensities, thereby yielding a faster temporal-spatial summation on cortico-motoneuron connections. We have also taken into consideration that some studies suggest the use of even higher intensities (some used 110–120% in individuals with Parkinson’s disease, for example) considering the elevated motor threshold of lower-limb muscles [33, 34]. The intensity of 100% was then chosen according the safety criteria suggested by Rossi et al. [9].

#### rTMS-sham

The rTMS-sham will be performed over five consecutive sessions per week (one session daily.) The sham coil will be used because it ensures the attenuation of the magnetic field while appearing to be the same shape as the active coil, with good approximation of auditory feedback [35]. In addition, the tactile contact of the coil with the skull is maintained.

### Procedures

The participants will be positioned comfortably in either a normal chair or a wheelchair, depending on the level of motor commitment of each patient. The feet will be positioned flat on the floor and the hands will rest on the thigh, in the supine position.

The rTMS stimulator will be connected to a figure-eight coil (i.e. butterfly coil) and positioned on the vertex of the lower-limb motor area, which corresponds to the apical surface of the skull. We chose to stimulate the vertex point because the motor impairment of subjects with iSCI occurs bilaterally, although the degree of involvement varies from one side to the other. Therefore, both cortical sides will be stimulated simultaneously, as the vertex point is equidistant between the left and right hemispheres [36, 37]. Standardized caps will be used, in accordance with the International 10-20 system EEG. Such a procedure will be used to find the motor threshold, a point which corresponds to the motor area of the *abductor pollicis brevis*, and so marks the point corresponding to the vertex.

### rTMS protocol

#### Magnetic stimulator

The rTMS will be applied using *Neurosoft - Neuro-MS 5* (Neurosoft Ltd®, Ivanovo, Russia), a commercially available transcranial magnetic stimulator equipped with an angulated figure-eight-shaped coil (AFEC – 01-100).

### Target

rTMS will be applied to an angulated figure-eight coil over the lower-limb motor area localized in M1 (in order to stimulate both lower limbs), with the handle of the coil parallel to the interhemispheric midline (pointing occipitally) as used by Khedr et al. [33], Jetté et al. [38], and Ji et al. [39] based on the vertex position of the International 10-20 system EEG.

When the orientation of the handle of a figure-eight coil is parallel to the interhemispheric midline (posterior–anterior direction), there is a TMS motor cortex activation through the preferential recruitment of cortical interneurons and through activating the pyramidal tract indirectly [17, 34, 40, 41]. Thus, the biological effect depends on the neuronal circuit finally recruited [17].

### rTMS sessions

Each session will be held at the Neuromodulation Laboratory of UEPB, using the same equipment during the same time of day. Each session will consist of 12 trains of 50 magnetic pulses at 5 Hz on each train. These pulses will be separated by 10-s intervals between each train. Intensity will be set at 100% of the individual’s RMT, defined as the lowest stimulation intensity that, within ten trials, induced at least five motor-evoked potentials assessed on the first dorsal interosseous muscle in the resting state. The parameters of rTMS were chosen based on safety parameters of the both National Institute of Neurological Disorders and Stroke (NINDS) and the paper by Rossi et al. [9].

### Primary outcomes

To evaluate the motor and sensory effects of the high-frequency rTMS in the lower-limb motor area on M1 (i.e. vertex, Cz) and check the possibility of generating motor gains in participants with iSCI, we will observe the change from baseline motor values provided by the ASIA score.

Only one researcher will be responsible for evaluating all of the participants through both the sensorimotor scales and electromyography (EMG). This researcher will be a physiotherapist who will also receive training to use the scales and EMG device. In addition, we will perform an evaluation of the intra-rater reliability of the instruments used in this study. Adverse events will be collected using a form for adverse events at each assessment point and any adverse event related to the intervention will be reported.

#### ASIA – motor score

The International Standards for Neurological Classification of Spinal Cord Injury (ISNCSI) is a medical examination from which part of the ASIA motor score is derived [42]. It uses a test of the strength of ten key muscles on each side of the body (e.g. elbow flexors, wrist extensors, hip flexors, quadriceps, dorsi flexors). The score ranges from 0 (no contraction) to 5 (normal resistance) through a full range of motion. A total possible score of 50 for the upper extremities (UE) and 50 for the lower extremities (LE) may be obtained [42].

### Secondary outcomes

#### ASIA – sensory score

The ASIA sensory score is also part of the assessment for the ISNCSI [42]. The test involves pinprick and light touch sensation at key points representing each dermatome of the body, scored on a three-point scale (0, 1, and 2). Scores will be summed to give a total possible score of 224, where a higher score indicates better sensation than a lower score [42].

#### Fugl–Meyer scale for upper and lower members (motor part - FMS)

The FMS is an instrument used to evaluate body function impairment after stroke [43]. This scale assesses five domains: motor; sensory; balance; range of motion; and joint pain [43]. The UE (i.e. shoulder, elbow, forearm, wrist, and hand) and LE (i.e. hip, knee, and ankle) are assessed within the motor domain, then impairment severity and functional ability are indexed [44]. Sullivan et al. [44] affirm that it is likely that the FMS motor score may be a clinical measure indicative of white matter damage within CST fibers. This potential can justify its use in SCI populations. This scale is a 226-point multi-item Likert-type scale used to measure recovery. Multiple items are scored on a three-point ordinal scale (0 = cannot perform, 1 = perform partially, 2 = performs fully) [43]. In addition, the motor domain includes score ranges from 0 (plegia) to a maximum of 100 (normal motor performance); 66 points can be divided for the UE and 34 points for the LE [44, 45]. In this study we will only use the motor domain and evaluation will take place bilaterally.

#### Surface electromyography (EMG)

The EMG will be recorded differentially using round-shaped surface electrodes (Ag-AgCl, 0.8 cm diameter, with an inter-electrode distance of 2 cm) over the following muscles: vastus medialis; vastus lateralis; rectus femoris; biceps femoris; gastrocnemius; and tibialis anterior. We will position the electrodes at standard Surface ElectroMyoGraphy for the Non-Invasive Assessment of Muscles (SENIAM) positions [46], and a ground electrode will be over the tibia of the left leg. The EMG signals will be amplified and filtered (5 Hz to 2 kHz) by a biological data acquisition system Miotool 400 (Miotec®, Brazil) and sent to an A/D interface (National Instruments, Austin, TX, USA) with a 2-kHz sampling rate. Data will be stored on hard disk for later off-line processing. Data will be collected using the software *Miograph* (*Miotec*®). The EMG acquisitions will be obtained during knee extension, knee flexion, plantar flexion, and dorsiflexion. For 1 min, participants will perform contractile movements with speed controlled by audible and visual signals. The normalization procedure for peak contraction will be made to standardize EMG acquisitions and make them comparable.

#### Modified Ashworth Scale (MAS)

The MAS is an instrument used to evaluate the spasticity (i.e. grading the resistance encountered during such passive muscle stretching) [47, 48]. The grades of spasticity are 0 (normal muscle tone), 1 (slight increase in muscle tone, when move a limb), 2 (more marked increase in muscle tone, but limb easily flexed), 3 (considerable increase in muscle tone), and 4 (limb rigid in flexion or extension) [47].

#### Socio-demographic questionnaire (SDQ)

The SDQ will be used to collect personal, socio-environmental, and clinical information. It will also determine information used to evaluate participants’ eligibility criteria. Our research center standardized this qualitative instrument, but a pilot test will be conducted to identify possible necessary adjustments to this questionnaire. In summary, this scale will be used as a sample description and as an eligibility criterion.

#### Scale of Mini-Mental State Examination

The MMSE assesses the mental state of the individuals assessed [49, 50]. The maximum score is 30 and there are two domains. The first tests for vocal responses, which covers orientation, memory, and attention (maximum of 21 points). The second tests for the ability to name objects, obey verbal and written commands, write a sentence spontaneously, and copy a complex polygon (maximum of nine points) [50, 51]. In Brazil, Bertolucci et al. [52] translated the MMSE. They found that the educational level of participants influenced their total score on the MMSE. As a result, the authors have proposed different cut-offs for the diagnosis of cognitive decline. The suggested cutoff points were 13 for illiterates, 18 for low and middle school, and 26 for high school. We will use this scale as an eligibility criterion.

#### Hamilton Rating Scale for Depression

The HAM-D is used to identify depression and contains 17 items plus four additional variables (diurnal variation, derealization, paranoid symptoms, and obsessive symptoms) [53]. Hamilton has not established a cut-off point, but in clinical practice scores higher than 25 points characterize severely depressed patients; scores in the range of 18–24 indicate moderate depression; and scores in the range of 7–17 indicate mild depression [54]. We will use this scale as an eligibility criterion.

#### Electroencephalogram

EEG provides a measurement of brain electrical activity (i.e. the amount of synaptic activity synchronized) recorded from scalp electrodes [55]. Applications of EEG can be used in identification of nature, type, and severity of epileptiform activity [56]. In this study, the EEG will be used to identify individuals with epilepsy or brain-activity patterns that may indicate possible underlying seizures. Therefore, this exam will serve only as an eligibility criterion.

#### Neurological examination (NE)

Clinical data collection will be achieved via qualitative standardized NE. This instrument consists of four parts: a history of the lesion and clinical symptoms; sensory and motor evaluation; functional activities; superficial and deep reflections.

### Statistical analysis

#### Sample size

The sample size was calculated using statistical software (GPower 3.1.5) [57] on the main outcome measure (i.e. the motor score). This calculation was based on data from one study with a group of SCI individuals that received high-frequency rTMS at a frequency of 5 Hz on the vertex, which related to an improvement in clinical measures of the motor score [24]. The power was 0.80; the alpha was 0.05, the effect size was 0.65 (Cohen’s *d*). The sample estimation indicated that 14 participants would be necessary (i.e. seven per group). With an adjustment to allow for a dropout rate, we will recruit 20 participants.

#### Data analysis

The analysis will follow a pre-specified analysis plan, based on comparing the groups as randomized (intention-to-treat). All data will be presented as mean ± standard deviation and summarized in frequency tables. We will use Microsoft Office Excel for Windows (version 2013) to store the data and the Statistical Package for Social Sciences (SPSS-IBM®) version 21 for Windows to perform statistical analysis. The normality and homogeneity of all variables (i.e. primary and secondary outcomes) will be tested with the Shapiro–Wilk and Levene’s test, respectively. For non-parametric data, the Friedman test will be used followed by the Wilcoxon post hoc test. We will use the ANOVA two-way (2 × 4) for the inferential statistic of the parametric data, with Tukey’s post hoc tests. Significance level will be set as α ≤ 0.05 and the effect size will be calculated by eta-squared (*ŋ* ^*2*^).

### Expected risks

High-frequency rTMS is a safe non-invasive technique according to current knowledge [9, 58]. Some studies indicate that there may be weak tension headaches and muscle spasms experienced during rTMS [59]. Overall, in cases where contraindications or safety parameters were not maintained, serious adverse events such as seizures occurred [9]. According to Rossi et al. [9], a large number of individuals were subjected to studies with rTMS since 1998 (when the limit parameters by Wassermann et al. [58] were defined) and a small number of seizures were observed. Therefore, the risk of rTMS-induced seizures is considered very low. To avoid adverse effects and risks to the individual, the rTMS parameters used in the present trial will be within the safety limits set by the NINDS and by Rossi et al. [9], which were updated security settings for transcranial magnetic stimulation.

### Expected benefits

Intervention with high-frequency rTMS over the parameters established in this study may generate motor gains and consequent functional recovery in patients with iSCI. Motor gains and functional recovery might contribute to an improved quality of life and functional independence. Moreover, the participation of those in this study will contribute towards the construction of scientific knowledge on the use of rTMS in iSCI.

## Discussion

Individuals with iSCI show clinical symptoms which are associated with reduced quality of life and functional independence [60, 61]. The CNS may be able to recover naturally after injury due to plasticity mechanisms and reorganization of residual neural pathways [5]. However, natural recovery is limited and cannot be relied upon [6]. It is therefore plausible that stimulation as a rehabilitation technique may be crucial in enhancing CNS plasticity. High-frequency rTMS has emerged as a promising technique in stimulating neural reorganization and synaptic plasticity in cortical and subcortical networks, thereby affecting the descending control of spinal excitability [11, 12, 14, 34, 62, 63]. These mechanisms might accelerate the development of neural connectivity responsible for motor function improvement [12, 14, 22, 24, 63].

Although an adaptive reorganization involves the formation of new connections and restoration of pre-existing connections, studies that directly address quantitative parameters associated with neuronal repair are limited. This is because most of the outcome measures are not able to assess the neurophysiological substrates that directly contribute to functional recovery, such as specific mechanisms within the brain and spinal cord that influence the generation of the motor command to the affected limbs [63].

The protocol described herein is expected to be the first fully randomized controlled double-blind crossover trial to assess the effect sizes associated with rTMS intervention in participants with iSCI. We expect the outcomes of the present study to provide additional clinical evidence of the potential benefits of high-frequency rTMS applied to the lower-limb motor area to improve sensorimotor recovery and/or to reduce spasticity in patients with iSCI.

### Trial status

Participant recruitment started in May 2017 and is expected to end in November 2017. Study completion is estimated by May 2018.

## Additional file

Additional file 1: SPIRIT 2013 Checklist. rTMS study protocol. (DOC 122 kb)

## Abbreviations

ASIA: American Spinal Injury Association Impairment Scale
CAEE: Certificado de Apresentação para Apreciação Ética (in Portuguese)
CAPES: Coordination for Higher Education Staff Development (in Portuguese)
CNS: Central nervous system
CST: Corticospinal tract
EEG: Electroencephalogram
EMG: Electromyography
FAPESP: Foundation of Support for Research of São Paulo State (in Portuguese)
FMS: Fugl–Meyer Scale
HAM-D: Hamilton Rating Scale for Depression
iSCI: Incomplete spinal cord injury
ISNCSI: International Standards for Neurological Classification of Spinal Cord Injury
LE: Lower extremity
M1: Primary motor cortex
MAS: Modified Ashworth Scale
MMSE: Mini-Mental State Examination
NE: Neurological examination
NINDS: National Institute of Neurological Disorders and Stroke
rTMS: Repetitive transcranial magnetic stimulation
SCI: Spinal cord injury
SDQ: Socio-demographic questionnaire
SENIAM: Surface Electromyography for the Non-Invasive Assessment of Muscles
SPIRIT: Standard Protocol Items: Recommendations for Interventional Trials
SPSS: Statistical Package for Social Sciences
UE: Upper extremity
